# The interpretation of disease phenotypes to identify TSE strains in mice: characterisation of BSE using PrP^Sc^ distribution patterns in the brain

**DOI:** 10.1186/1297-9716-43-86

**Published:** 2012-12-17

**Authors:** Erica Corda, Katy E Beck, Rosemary E Sallis, Christopher M Vickery, Margaret Denyer, Paul R Webb, Susan J Bellworthy, Yvonne I Spencer, Marion M Simmons, John Spiropoulos

**Affiliations:** 1 School of Veterinary Medicine, University of Milan, Milan, Italy; 2 Animal Health and Veterinary Laboratories Agency, Woodham Lane, New Haw, Addlestone, Surrey, KT15 3NB, United Kingdom

## Abstract

In individual animals affected by transmissible spongiform encephalopathies, different disease phenotypes can be identified which are attributed to different strains of the agent. In the absence of reliable technology to fully characterise the agent, classification of disease phenotype has been used as a strain typing tool which can be applied in any host. This approach uses standardised data on biological parameters, established for a single host, to allow comparison of different prion sources. Traditionally prion strain characterisation in wild type mice is based on incubation periods and lesion profiles after the stabilisation of the agent into the new host which requires serial passages. Such analysis can take many years, due to prolonged incubation periods. The current study demonstrates that the PrP^Sc^ patterns produced by one serial passage in wild type mice of bovine or ovine BSE were consistent, stable and showed minimal and predictable differences from mouse-stabilised reference strains. This biological property makes PrP^Sc^ deposition pattern mapping a powerful tool in the identification and definition of TSE strains on primary isolation, making the process of characterisation faster and cheaper than a serial passage protocol. It can be applied to individual mice and therefore it is better suited to identify strain diversity within single inocula in case of co-infections or identify strains in cases where insufficient mice succumb to disease for robust lesion profiles to be constructed. The detailed description presented in this study provides a reference document for identifying BSE in wild type mice.

## Introduction

Transmissible spongiform encephalopathies (TSEs) are fatal neurodegenerative diseases that affect humans and animals. The hallmark lesion of TSEs is spongiform changes in the brains of affected subjects. According to the prion hypothesis TSEs are caused by proteinaceous infectious particles consisting of abnormal isoforms (PrP^Sc^) of the normal cellular prion protein (PrP^C^) [1-3] although this view is not accepted universally [4-6]. Despite the disagreement in the scientific community regarding the exact nature of the infectious agent, PrP^Sc^ is considered to be a ubiquitous marker of TSE disease and it accumulates in the brains of affected subjects from the very early stages of the disease before any spongiform changes become evident [7,8]. Therefore, techniques such as immunohistochemistry (IHC) are widely used to detect PrP^Sc^ as a marker of disease and diagnose experimental [7] or naturally occurring [9,10] TSEs.

Scrapie, the archetypal TSE which affects small ruminants, is not considered to be a public health risk and is known for centuries [11-13]. In contrast, bovine spongiform encephalopathy (BSE) is a TSE of cattle first recognised in 1986 [14]. After its discovery, novel spongiform encephalopathies in captive ruminants [15] and cats [16-18] and a variant of Creutzfeldt-Jakob disease (vCJD) in humans [19-21] were identified. Subsequently a link between BSE and these new emerging TSEs was established and wild type mouse bioassay contributed in determining this association [22].

Wild type mouse bioassay has been used to study the infectivity of TSE sources and to identify different strains of the agent [23-25]. According to established methodology, strains are identified after serial passage in a given mouse line to allow a TSE agent to adapt into the new host by overcoming the low attack rates, and prolonged and variable incubation periods which are experienced during primary isolation of the agent into mice, a property called the species or transmission barrier. For strain typing C57BL/6, RIII and VM are the most commonly used wild type mouse lines either alone or in combination [26].

The amino acid sequence of PrP in RIII and C57BL/6 mice (*Prnp*^*a*^) differs from that of VM mice (*Prnp*^*b*^) at codons 108 and 189 (*Prnp*^*a*^ – leucine, threonine; *Prnp*^*b*^ – phenylalanine, valine) [27,28]. This difference in *Prnp* genotype affects both bioassay parameters which are traditionally used to study TSEs in mice: the incubation period and the lesion profile – a semi-quantitative assessment of vacuolation at specific neuroanatomical areas [23,24,29]. However, there are some differences in the data observed between RIII and C57BL/6 mice challenged with the same TSE source or strain attributed to genetic factors other than the PrP genotype [30,31]. Also in RIII mice, even upon primary isolation, BSE and BSE derived TSEs behave reproducibly with consistent, albeit prolonged compared to serial passage, incubation period and exhibit a single characteristic lesion profile [22,32-34]. In contrast to RIII mice, primary isolation of BSE in C57BL/6 and VM mice does not yield informative data according to traditional parameters. However, as for classical scrapie, the agent can be fully characterised in these mouse lines upon serial passage. Incubation period and lesion profile data obtained from the serial passage of BSE through C57BL/6 and VM mice indicate that there are two stable mouse adapted strains of BSE: 301C which is derived after serial passage through C57BL/6 mice and 301V which results after serial passage through VM mice [35].

The association of vCJD with BSE [22,36,37], the successful experimental transmission of BSE to sheep [38,39] and the first report of BSE in a goat [40], raised the need to be able to confidently distinguish between BSE and scrapie, and applying traditional methods on RIII bioassays is regarded as one of the most reliable approaches to do so [22,32]. There are some limitations, however, as it has been demonstrated by recent studies, according to which scrapie-infected sheep can produce a similar incubation period and lesion profile to BSE upon primary isolation in RIII mice [34,41]. It was still possible, however, to confirm these cases as scrapie using histopathology, Western blot and IHC although detailed description of the PrP^Sc^ types in the mouse brain was not attempted. Other studies also indicate that an assessment of the distribution of PrP^Sc^ in the brain could aid discrimination of TSE source [42,43]. These data, although limited, suggest that assessment of PrP^Sc^ distribution could provide an alternative approach to lesion profiling in distinguishing BSE from non-BSE related TSEs upon primary passage and the potential to diagnose BSE based on individual mice rather than groups of animals makes this method very powerful as a discriminatory tool. This approach has recently been employed to help confirm BSE in a goat in UK where incubation period and lesion profile data on primary isolation were unavailable [44].

IHC is used routinely as a diagnostic tool in the study of TSEs in large animals. Furthermore, IHC has been used to characterise TSE sources within sheep by the identification of different PrP^Sc^ types and the spatial distribution of each type in the brain to give specific PrP^Sc^ distribution patterns [10,45-47]. It has been proposed that these patterns may be dependent on agent strain, host genetic parameters (the most profound of the host factors being its PrP sequence) or a combination of both [47]. In contrast, there is limited data available regarding the systematic observation of PrP^Sc^ patterns and their neuroanatomical distribution upon primary isolation in murine TSE studies. The majority of studies involve mouse-adapted scrapie strains, such as 87V or ME7, and a systematic recording of the different PrP^Sc^ types in the murine brain was not attempted [48-50].

In the current study we systematically studied and recorded in detail the distribution of PrP^Sc^ throughout the brain of wild type mice challenged with BSE from bovine and ovine sources during primary isolation or after serial passages using IHC and PET blot. We were able to identify source- and strain-specific markers, in addition to mouse lineage-specific markers which could enhance our ability to discriminate between TSE sources and help characterise strains on primary isolation.

## Materials and methods

### Inocula selection

In the current study we examined retrospectively, using IHC, selected BSE inocula which had previously been bioassayed into RIII mice and at least in C57BL/6 or VM mice. These inocula included five bovine BSE pools composed of either natural or experimental BSE cases, one ovine BSE pool and a single ovine BSE source. All sources had been characterised previously in RIII mice and their biological properties at primary isolation were consistent with BSE [33].

Murine brains from BSE inoculated mice that were diagnosed TSE positive at primary isolation were used to prepare inocula for serial passages. In addition 301C or 301V infected murine brain reference materials were also used for serial passages.

### Animal husbandry and inoculations

For primary isolation 5–10 week old mice were inoculated intracerebrally (20 μl) and intraperitoneally (100 μL) with brain homogenates (10% w/v in normal saline) from BSE affected cattle or sheep; each inoculum was administered to groups of 20 mice. For serial passages groups of 10 mice were inoculated intracerebrally with 20 μL of homogenate (1% w/v in normal saline) from BSE affected mice. All homogenates were microbiologically screened and treated with antibiotics if required. Only bacteriologically clear homogenates were used for inoculations.

Intracerebral inoculations were performed under general anaesthesia using a 25G hypodermic needle attached to a 1 mL insulin syringe. A plastic sheath was inserted along the needle allowing only the distal 5 mm of the needle to be exposed to ensure that the inoculum was deposited at similar depth in each animal and minimize tissue injury. The point of entry was approximately 3 mm dorsally from a point lying halfway between the eye and the base of the ear. Insertion into the CNS was achieved by gently rotating the syringe along its axis at right angle with respect to the skull surface.

Mice were housed in standard mouse cages and were monitored for clinical signs of disease by experienced animal attendants. Mice were euthanized after exhibiting clinical signs of TSE disease for two consecutive weeks unless the clinical progression of the disease was so rapid that they had to be euthanized sooner on welfare grounds. All mice that did not show clinical signs of TSE were allowed to live until they were euthanized on welfare grounds caused by other conditions (intercurrent deaths).

All animal procedures were performed in compliance with the Animal (Experimental Procedures) Act 1986 under license issued by the UK Home Office and were approved by the Ethics Review Committee.

### PrP-immunohistochemical (IHC) and Paraffin Embedded Tissue (PET) blot analysis

After euthanasia the brain of each mouse was removed aseptically and sectioned parasagitally. Approximately one third was stored at −80°C for bioassay or biochemical studies and two thirds were immersed in buffered formalin for 48–72 h at room temperature. At the end of the fixation the samples were sectioned at 4 different coronal levels at the level of medulla (including the cerebellum), midbrain, thalamus (including hippocampus and overlying cortex) and frontal cortex (including basal ganglia) as described elsewhere [33].

Each coronal segment was embedded in paraffin wax and histological sections (3 μm thick) from each level were mounted on positively charged slides and were subjected to IHC using the rabbit polyclonal anti-PrP antibody Rb486 [51].

Sections (3 μm thick) of samples that were analysed with IHC were also processed fro PET blot using the mouse monoclonal antibody 2 G11 as described previously [52,53].

## Results

To confirm that the selected inocula were characteristic of BSE, the lesion profile data from RIII mice were analysed. The typical BSE associated lesion profile which is characterised by peaks at grey matter areas G1 (dorsal medulla nuclei), G3 (cortex of the superior colliculus) and G7 (septal nuclei of the paraterminal body) was observed in all selected cases [22,32,33]. The attack rate, incubation period and Western blot data generated by these inocula in RIII mice were also consistent with BSE (data not shown) suggesting that the selected sources demonstrated typical BSE behaviour.

### Identification and terminology of PrP^Sc^ types

A number of PrP^Sc^ types were observed in BSE-infected mice. Different PrP^Sc^ types were identified and the following terminology was used throughout the current study to describe the observed PrP^Sc^ types in BSE-infected mice.

#### Granular

**Figure 1:**
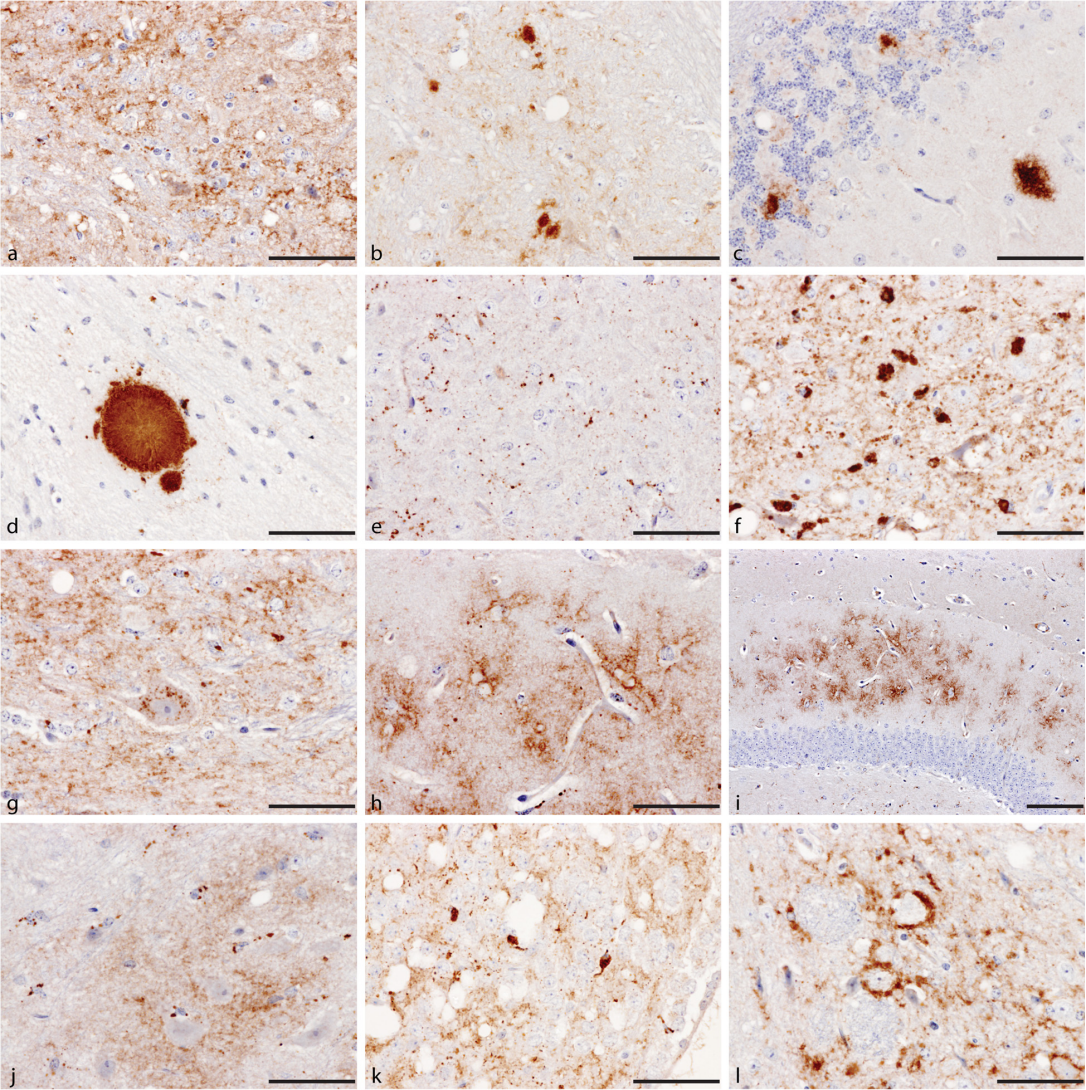
**PrP^Sc ^types identified in wild type mice infected with BSE.** For definition of each type refer to the text. PrP^Sc^ types shown are (**a**) granular, (**b**) small and medium aggregates, (**c**) medium and large aggregates, (**d**) fibrillar plaque, (**e**) intraglial (**f**) speckled, (**g**) intraneuronal, (**h** and **i**) stellate, (**j**) intraglial, (**k**) larger intraglial deposits observed in the habenular nucleus of VM mice and (**l**) perineuronal. Scale bar represents 50 μm except in (**i**) where it represents 100 μm.

Granular type appeared as small particulate PrP^Sc^ deposits with indistinct borders that appeared to diffuse in the neuropil (Figure 1a).

#### Aggregates

Focal dense deposition of PrP^Sc^ in the neuropil which varied in dimensions (Figure 1b-c).

#### Plaques

Plaques appeared as discrete, organised deposits of PrP^Sc^ with a central core surrounded by radiating fibrils within the neuropil (Figure 1d). They were not encountered very often and therefore cannot be considered a consistent feature of the IHC phenotype in BSE infected wild type mice. Strict criteria were applied for the characterisation of plaques: non fibrillar formations that lacked a central core were classified as aggregates even if they had a distinct circular or elliptical border and a relatively organised structure. Such structures may result from real plaques if the sectioning plane is distant from the central core of the plaque. Alternatively they may represent plaques at early stages of their formation before they develop fully characteristic plaque features. The appearance of plaques was not consistent or unique to BSE and therefore their significance as a discriminating parameter was limited.

#### Punctate

Punctate deposits appeared as discrete, small particles of PrP^Sc^ within the neuropil (Figure 1e). Occasionally, this type of deposit tended to align to give a beaded linear appearance.

#### Speckled

Speckled type appeared as clustered dense deposits, distinct from aggregates and plaques, and can be associated with glial cells (Figure 1f).

#### Intraneuronal

Intraneuronal type appeared as multiple small, discrete granules of PrP^Sc^ within the cytoplasm of larger neurons. It was observed in targeted nuclei that usually house large neurons, such as locus coeruleus, cerebellar and red nuclei (Figure 1g). The appearance of this feature was not consistent or unique to BSE and therefore its significance as a discriminating parameter was limited.

#### Stellate

Stellate type appeared as small, numerous radiating “star-like” deposits of PrP^Sc^ within the neuropil (Figure 1h) and was easier to identify at lower magnification (Figure 1i). The designation of this term was based purely on morphological criteria although in ruminants this term has been associated with glial cells.

#### Intraglial

Intraglial type appeared as dense deposits in close proximity to the nuclei of glial cells (Figure 1j). As with the stellate type, this is also an operational term indicating the common features of these deposits with the intraglial deposits observed in TSE affected ruminants. In VM mice an intraglial type comprising larger deposits was also observed more consistently in the habenular bodies (Figure 1k).

#### Perineuronal

Perineuronal type appeared as deposition of PrP^Sc^ around neuronal perikarya. (Figure 1l). As with the intraneuronal type the significance of perineuronal type as a unique BSE feature was restricted, and therefore the significance of this PrP^Sc^ type as a BSE trait was limited.

In contrast to standard TSE lesion protocols where 9 grey matter and 3 white matter brain areas are analysed, it was necessary and advantageous to examine all neuroantomical areas presented in each coronal section when mapping PrP^Sc^ types. The consideration of specific PrP^Sc^ types in exact neuroanatomical structures facilitated the construction of PrP^Sc^ distribution patterns.

### PrP^Sc^ distribution patterns resulting from primary isolation of BSE in C57BL/6 and RIII mice

Inoculation of BSE into C57BL/6 and RIII mice resulted in very similar patterns which was expected as these two mouse lines share the same PrP genotype. Therefore the description of the PrP^Sc^ patterns is based on observations in C57BL/6 mice and any differences in the RIII mice are indicated.

#### Medulla section

Granular deposits were detected throughout the medulla with varying intensity; occasionally small to medium aggregates were also dispersed throughout the medulla. This pattern was observed with higher intensity in the cochlear nuclei. Intraglial and intraneuronal types were also observed but not consistently and their presence in the medulla is not considered to be a strong discriminatory characteristic of BSE in these mice. In the locus coeruleus and the cochlear and cerebellar nuclei the intensity of the immunolabelling was greater than in the rest of the medulla. Similar observations were made in the medullae of RIII mice although the immunolabelling was less intense.

The granular and the molecular layers of the cerebellum in mice with BSE characteristically displayed widespread granular deposits and aggregates ranging from small to large. In RIII mice this pattern was less intense and the molecular layer was rarely populated with PrP^Sc^ deposits.

#### Midbrain section

Granular deposits were observed throughout the midbrain accompanied by small to medium aggregates and low level intraglial labelling. The raphe and red nuclei and, in RIII mice the pars compacta of the substantia nigra, usually appeared more prominent, because this pattern became denser, with the presence in the red nuclei of bigger aggregates and, in C57BL/6 mice of intraneuronal PrP^Sc^. A distinctive characteristic of the RIII mice was that the superior grey layer of the superior colliculus appeared spared of PrP^Sc^ deposition at low magnification.

#### Thalamic and frontal sections

At the thalamic level the hypothalamus, especially the dorsomedial nuclei, displayed granular type with small aggregates distributed unevenly. Granular deposits were also observed in the habenular bodies and in the paraventricular nucleus of the thalamus. In C57BL/6 mice the zona incerta was usually easily distinguishable, due to the presence of a granular deposits, small to medium aggregates and sometimes perineuronal type. Widespread granular type was observed in the CA2 and, to a lesser extent, in the CA1 areas of the hippocampus, both in C57BL/6 and RIII mice. In some mice small aggregates located near the hippocampal fissure were also observed. Occasionally, plaques would be observed mainly along the corpus callosum and the external capsule.

In the cerebral cortex granular deposits were distributed along the internal pyramidal layer (layer V) with varying intensities spatially; in RIII mice this labelling appeared less intense overall. In the dorsolateral septal nuclei the granular deposits were milder and more evenly distributed. Granular deposits were widespread in the caudate putamen and in C57BL/6 mice this area also hosted small PrP^Sc^ aggregates.

**Figure 2:**
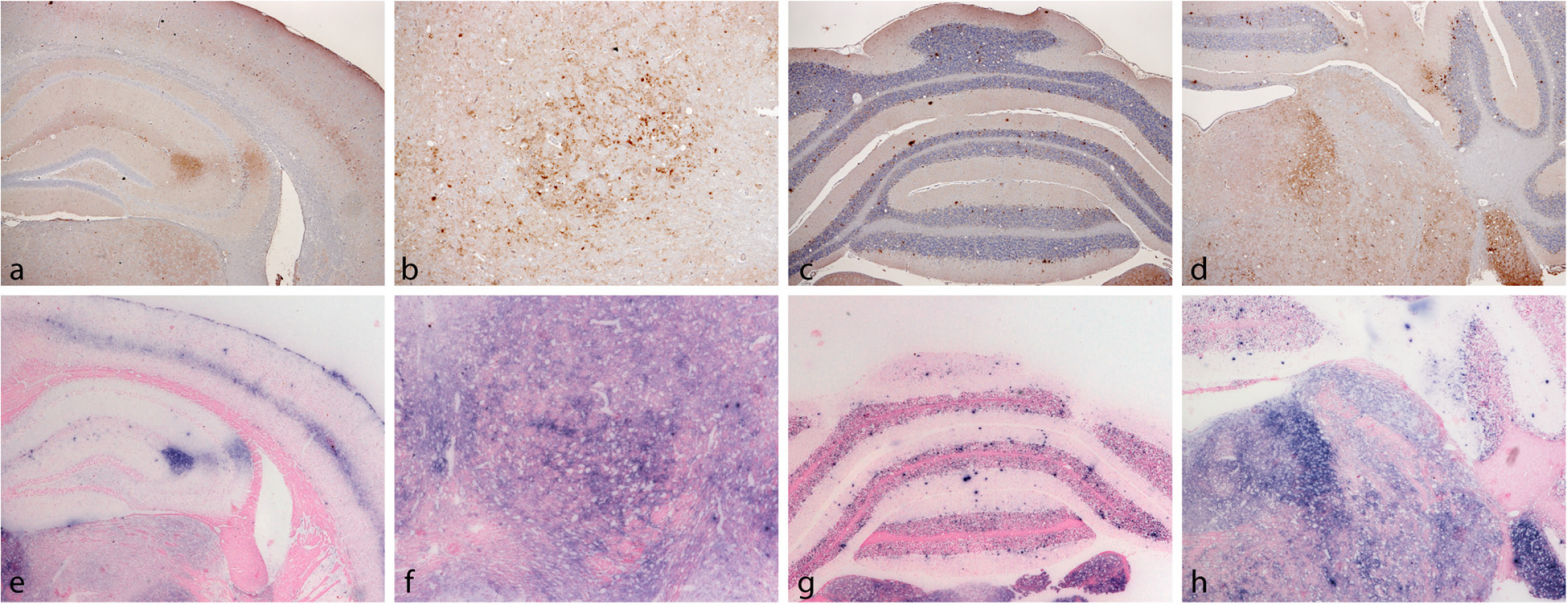
**Some distinctive features of BSE in C57/BL6 mice.** (**a**)-(**d**) Immunohistochemistry showing characteristic features in CA2 area (**a**), red nucleus (**b**), cerebellum (**c**) and the increased intensity of labelling in locus coeruleus and cochlear nucleus in the medulla (**d**). (**e**)–(**h**) The same areas stained using the PET-blot approach. Both methods revealed similar distribution of PrP^Sc^ but IHC confered higher resolution.

Characteristic aspects of this pattern which could help identify BSE in a single *Prnp*^*a*^ mouse using IHC or PET blot are presented in Figure 2.

### PrP^Sc^ distribution patterns resulting from primary isolation of BSE in VM mice

#### Medulla section

Punctate deposits were distributed throughout the medulla which was also affected by granular deposits of varying intensity where small aggregates, intraglial and intraneuronal PrP^Sc^ types were dispersed. This pattern became more intense in the locus coeruleus and the cerebellar and cochlear nuclei, although in this last area no intraneuronal deposition was observed. In addition, the locus coeruleus was the only neuroanatomical area where it was possible to observe the speckled PrP^Sc^ deposits, a distinctive feature of the VM mice. The nucleus of the solitary tract appeared relatively spared of granular type compared to the neighbouring areas, displaying only punctate PrP^Sc^ deposition.

In the granular layer of the cerebellum punctate and granular types were distributed unevenly with slightly increased labelling in the white matter border. The white matter of the cerebellum was also affected, mainly with granular deposits. In the molecular layer granular deposits appeared infrequently.

#### Midbrain section

In the midbrain the peri-aqueductal grey matter characteristically appeared spared of granular type compared to the surrounding areas, displaying only a punctate PrP^Sc^ deposition as was also observed in the midline raphe and the solitary tract. The rest of the mid-brain presented granular and intraglial deposits with varying intensity whilst punctate deposits and small aggregates were observed throughout the section. In the substantia nigra, the interpenducular and red nuclei the intensity of the staining was increased compared to the rest of the midbrain.

#### Thalamic and frontal sections

At the thalamic level the habenular bodies, thalamus, lateral thalamus, laterodorsal thalamic nuclei and zona incerta displayed granular and punctate types thoroughout, with small aggregates and intraglial type appearing multifocally. Intraglial deposits were particularly prominent in the habenular bodies. On the contrary, the hypothalamus and lateral hypothalamus were characterised by the presence of punctate deposition alone, which occasionally aligned to form beaded linear PrP^Sc^ deposits. Occasionally the arcuate thalamic nuclei and the median eminence displayed granular deposits and aggregates.

Punctate type was evident in the hippocampus and the dentate gyrus. In addition stellate deposits could be observed in the dentate gyrus, a characteristic which became more prominent and extensive after serial passage. Occasionally, plaques would be observed in the hippocampal molecular layer in the vicinity of the corpus callosum and the external capsule.

In the internal pyramidal layer of the cerebral cortex granular PrP^Sc^ deposition of variable intensity which made it appear mottled was recorded. The same areas were affected by intraglial deposits. Punctate deposition was predominant in the vertical limb of the diagonal band, medial preoptic band, ventral pallidum and dorsolateral septal nuclei. The dorsolateral septal nuclei were also affected by weak granular deposits. On the contrary, in the caudate putamen there were low levels of granular and punctate deposits.

**Figure 3:**
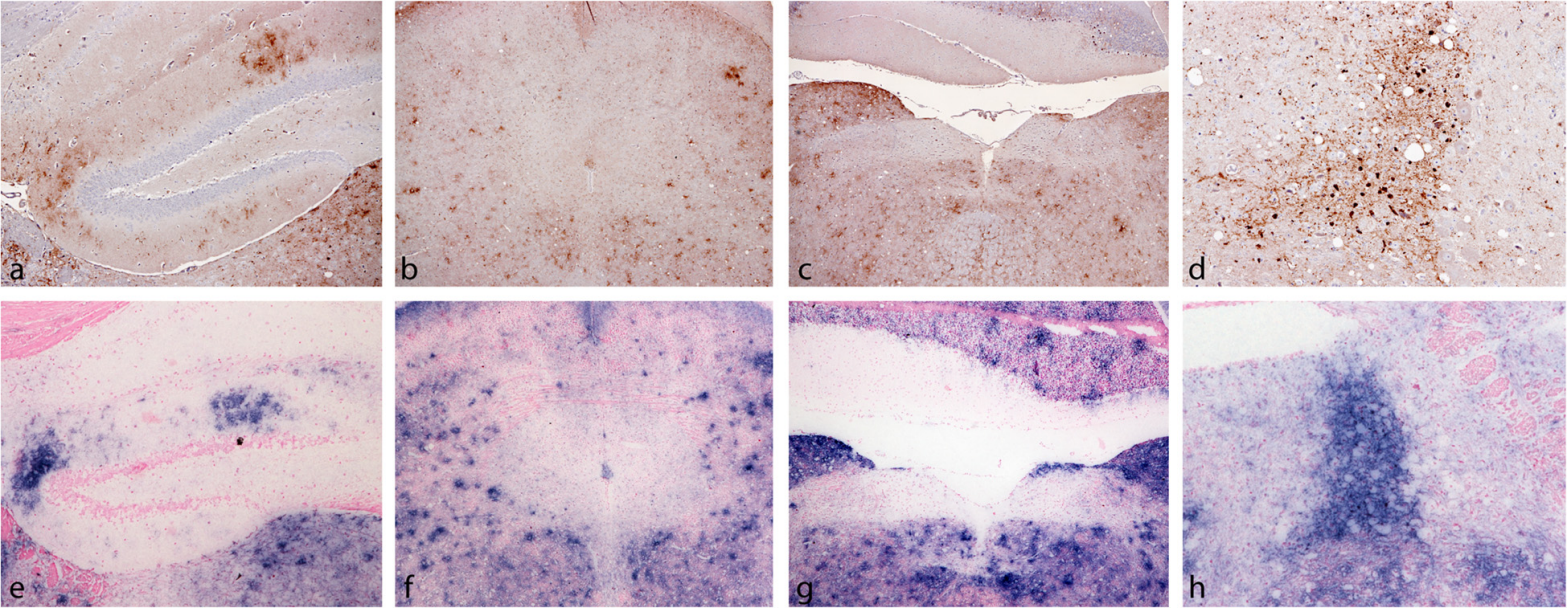
**Some distinctive features of BSE in VM mice.** (**a**)-(**d**) Immunohistochemistry showing characteristic features in the dentate gyrus (**a**), periaqueductal grey mater (**b**), solitary tract (**c**) locus ceruleus (**d**). (**e**)–(**h**) The same areas stained using the PET blot approach. Both methods revealed similar distribution of PrP^Sc^ but IHC confered higher resolution.

Characteristic features of this pattern which could help identify BSE in a single *Prnp*^*b*^ mouse using IHC or PET blot are presented in Figure 3.

Primary isolation of ovine BSE demonstrated that the distribution of PrP^Sc^ types in all mouse lines were indistinguishable from those observed for bovine BSE or for BSE derived from other sources (data not shown). In addition comparative studies with the anti-PrP antibody 2 G11 (data not shown) indicated that the PrP^Sc^ distribution patterns detected were indistinguishable from that observed with Rb486 suggesting that the patterns identified were not antibody dependent but a feature of the strain-host combination.

### PrP^Sc^ distribution patterns resulting from BSE serial passages in C57BL/6 and VM mice

Between first and second passage of BSE in C57BL/6 mice only subtle differences were introduced. They included a wider distribution of granular deposits and small aggregates in the hippocampus along the hippocampal fissure and occasionally in other layers whilst in mice challenged with ovine or bovine BSE the PrP^Sc^ appeared to be more restricted in the hippocampal fissure and the CA2 area of the hippocampus. In the cerebral cortex, in the proximity of the cingulum, a partial double layer of granular PrP^Sc^ was observed which appeared to overlay mainly along cortical layer V and partially along the multiform layer (layer VI) in its vicinity with the white matter - a distinguishing feature from primary isolation of BSE where PrP^Sc^ was more restricted giving the appearance of a single layer of granular deposits overlaying mainly cortical layer V. The remaining characteristic features of BSE were maintained after serial passage. The pattern observed at the second passage remained unchanged after subsequent serial passages of BSE isolates and was indistinguishable from the pattern produced by reference strain 301C.

**Figure 4:**
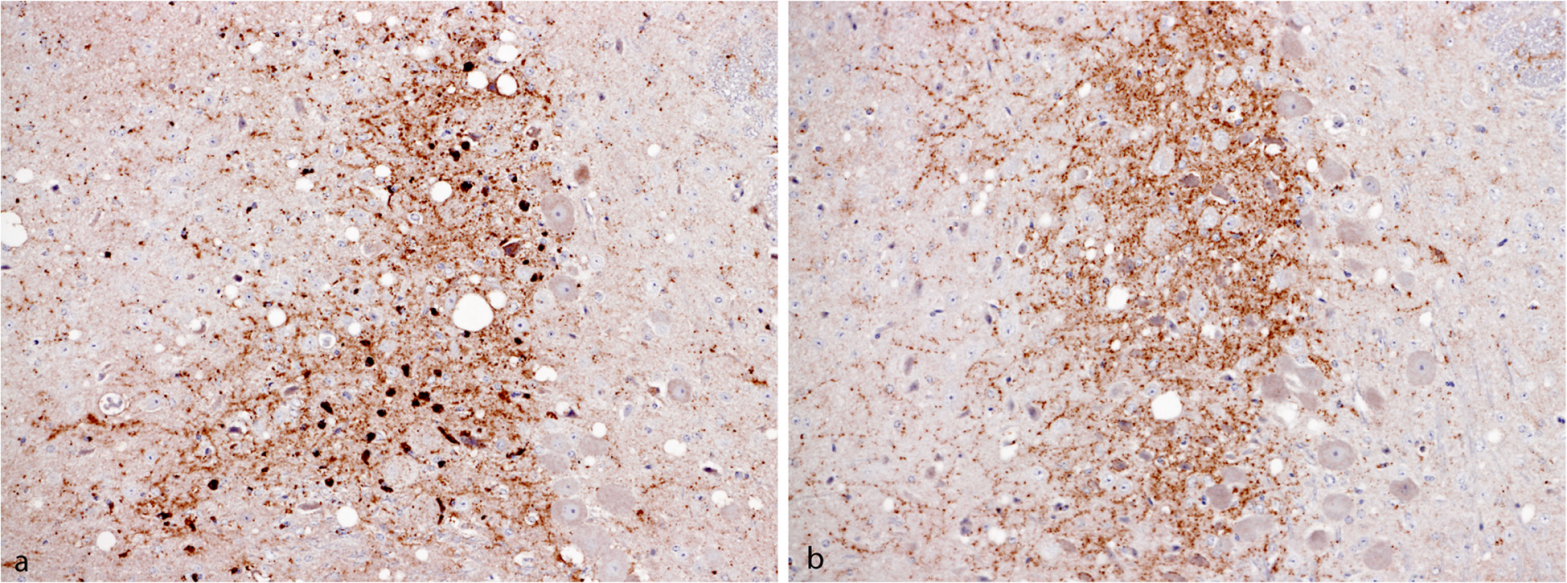
**Locus coeruleus of VM mice challenged with BSE.** (**a**) Primary isolation; (**b**) second passage. The characteristic speckled PrP^Sc^ deposits are evident only on primary passage.

The most significant feature between first and second passage of BSE in VM mice was a lack of appreciable punctate PrP^Sc^ deposits. Also the speckled deposits in the locus coeruleus were present only on primary isolation (Figure 4). Stellate PrP^Sc^ deposits became more defined and, in addition to the dentate gyrus, they were also observed in the hippocampus, the cerebral cortex along layer V and the layers underlying the meninges and the subiculum. In many of these areas during primary passage the main PrP^Sc^ type was granular with uneven intensity suggesting a pattern evolution. Other characteristic features of serially passaged BSE were reminiscent of primary isolation including a significant decrease of labelling in the nucleus of the solitary tract, raphe nuclei and peri-aqueductal grey matter, with evident PrP^Sc^ in surrounding areas. As in the C57BL/6 mice this pattern also remained unchanged after repeated serial passages of BSE isolates in VM mice and was indistinguishable from the pattern generated by reference 301V.

## Discussion

In this study we showed that after first passage of BSE in C57BL/6, RIII or VM mice a number of PrP^Sc^ specific deposits can be identified and their distribution in the murine brains can generate high resolution patterns. These distribution patterns were consistent and identifiable in individual mice. Although we only report here on BSE generated patterns we are aware that these patterns differ from the patterns produced in these mouse lines by natural cases of classical scrapie [34,41,53,54]. Therefore IHC can be used to identify TSE agents isolated in murine bioassays and facilitate resolve ambiguous TSE cases. Indeed this approach has been used successfully in two recent reports to help reject the presence of BSE in two sheep in one and confirm it in a goat in another [41,44].

Stabilization of a TSE agent in a wild type murine host requires serial passages within this host which have a significant effect on incubation period and lesion profiles [26,41]. However, PrP^Sc^ patterns are affected little and in a predictable way by serial passages [41,55,56]. This biological property makes PrP^Sc^ deposition pattern mapping a powerful tool in the identification and definition of TSE strains on first passage [34,41,44,54]. It can be applied to individual mice and therefore it is better suited to identify strain diversity within single inocula in case of co-infections [41,56]. It can also help substantially with the characterisation of cases in which insufficient mice succumb to disease for robust lesion profiles to be constructed [44].The detailed description presented in this study will hopefully provide a reference document for identifying BSE in wild type mice. In addition it provides a methodological approach which can be applied in other experimental rodents either wild type or transgenic to help identify TSE strains on primary isolation.

It is important to realise that it is not a requirement that all strain specific markers or the entire pattern are present in an individual mouse, although the majority will be. However, conclusions should not be based on single individual markers as some can be associated with other strains. For example, although in C57BL/6 mice the granular deposits in the CA2 area of the hippocampus are considered to be a BSE feature they can also be induced by 221C, a murine classical scrapie strain. Nevertheless, distinct differences in other markers can make distinction between these two strains feasible [53]. Therefore, in order to conclude that a pattern in an individual mouse signifies BSE or any other TSE strain it is important that more than one individual IHC markers which are compatible with a pattern produced by a specific TSE strain are identified; usually 3 preferably 4 characteristic IHC markers are adequate for strain identification in an individual mouse. The presence of a single pattern with the addition of uncharacteristic markers reminiscent of other strains or of elements of two patterns may signify coinfection of two strains as previously suggested [41,56].

Our observations are in agreement with the findings of a previous report [43] especially with regard to the distribution of PrP^Sc^ in the cerebellum and hippocampus. However, our study is more detailed in terms of the different PrP^Sc^ types and their distribution to specific neuroanatomical locations. Although IHC has also been applied to describe PrP^Sc^ patterns in murine adapted TSE strains [49,50,57,58] the potential of this technique as a powerful phenotypic tool that can be used to discriminate TSE strains not only after adaptation in the murine host but even at primary isolation has not been realised until recently [34,41,44,54,59,60]. This is intriguing because PET- or Histo- blots have been used to characterise successfully TSE strains during primary isolation although they have lower resolution compared to IHC as they only portray general PrP^Sc^ distribution [42,61]; at this level of resolution IHC and PET- or Histo-blots can be directly comparable and have been used to show strain similarities [60].

In conclusion the current study utilised IHC to successfully identify PrP^Sc^ patterns for BSE and BSE-derived strains in wild type mice. In a similar study we have identified PrP^Sc^ patterns in wild type mice that are associated with classical scrapie strains [52]. There is no resemblance between any of the classical scrapie and BSE patterns identified suggesting that PrP^Sc^ patterns in the brain of wild type are strain specific and can be applied even at primary isolation to provide a powerful tool to aid TSE strain discrimination in individual animals in association with other phenotypic parameters such as Western blot.

## Competing interests

The authors declare that they have no competing interests.

## Authors’ contributions

EC analysed IHC data, drafted figures and the manuscript. KB RS and CMV analysed IHC data and helped to draft the manuscript. MD and PW performed IHC and PET-blots. SB provided samples and participated in experimental design. YS provided technical expertise and participated in experimental design. MMS provided interpretational pathology expertise and participated in experimental design. JS participated in IHC and PET blot data analysis, provided interpretational pathology expertise, designed and coordinated the work and helped to draft the manuscript. All authors reviewed and approved the final manuscript.
